# Supplementary material for: Improving Multi-Tumor Biomarker Health Check-Up Tests with Machine Learning Algorithms
Source: Cancers (Basel). 2020 Jun 1;12(6):1442. doi: 10.3390/cancers12061442 (PMC7352838; doi:10.3390/cancers12061442)
Supplement: Supplementary file 1 [file cancers-12-01442-s001.pdf]

Supplementary Materials:

Improving Multi-Tumor Biomarker Health Check-up Tests with Machine Learning Algorithms

Hsin-Yao Wang, Chun-Hsien Chen, Steve Shi, Chia-Ru Chung, Ying-Hao Wen, Min-Hsien Wu, Michael S. Lebowitz, Jiming Zhou and Jang-Jih Lu

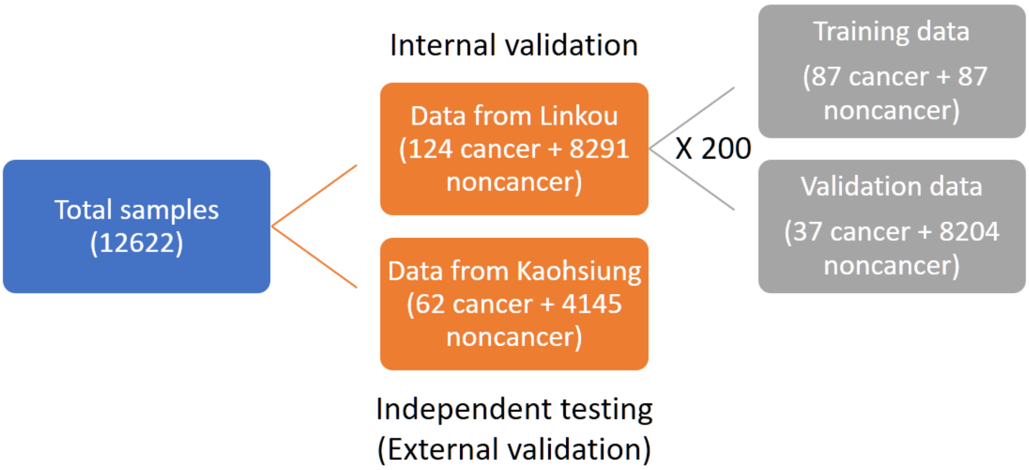

Figure S1: Data preparation shown here with male as an example, and female has the same structure. For each subsampling, we first split the cancer cases into training and validation datasets by ratio 70:30; for training dataset, we randomly took the same number of non cancer cases from the whole non cancer cases in Linkou branch, and the rest of the non cancer cases went to validation data. Thus, for male model, the training data had 87 cancer cases and 87 non cancer cases, the validation data had 37 cancer cases and 8204 non cancer cases, whose cancer versus non cancer case ratio remained as the same as the original dataset from Linkou branch After the training and internal cross validation for 200 times, we took all the 124 cancer cases and randomly selected 124 non cancer cases to build the cancer screening ML models. The data collected from Kaohsiung branch were used as the independent testing dataset to test the robustness of the ML models.

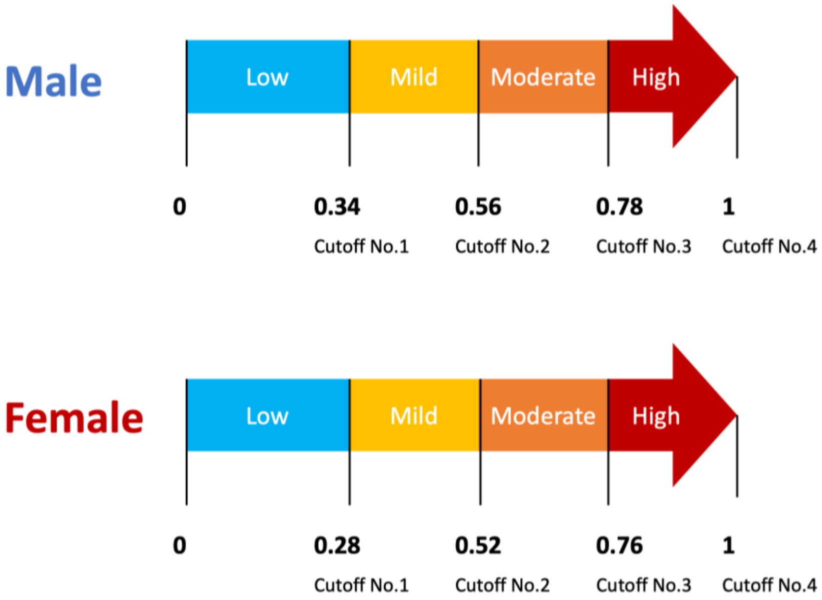

Figure S2. The cutoffs for males (LR algorithm) and females (RF algorithm).

Table S1. Organ system clustering and labelling.

Organ System Label	Female	Male
**General Surgery**	Breast cancer, Thyroid cancer, and Liposarcoma	NA
**Chest**	Lung cancer	Lung cancer
**Dermatology**	Skin cancer	Skin cancer
**Ear, Nose, and Throat**	H&N cancer, parotid cancer	H&N cancer, thyroid cancer
**Gastrointestine**	HCC, CRC, Gastric cancer, Pancreatic cancer, Esophageal cancer, Gallbladder cancer	HCC, CRC, Gastric cancer, Pancreatic cancer, Esophageal cancer, Gallbladder cancer
**Genitourinary**	Bladder cancer, RCC	Prostate cancer, Bladder cancer, RCC
**Hematology**	Leukemia, Lymphoma	Leukemia, Lymphoma
**Neurology**	CNS cancer	CNS cancer
**Gynecological**	Cervical cancer, Ovarian cancer, Uteral cancer	NA

Table S2. Performance comparison for cancer screening. Interpretation of tumor marker values used for cancer screening with the machine learning algorithms outperforms current interpretative criteria (i.e., a single threshold method; combined test of tumor markers). Machine learning models using LR algorithm and RF algorithm outperforms other algorithms in males and females, respectively.

Male	LR	Single Threshold Method
**AUROC**		
**Internal CV**	**0.7654 (0.7596, 0.7713)**	**0.6581 (0.5930, 0.7231)**
**External validation**	**0.8736 (0.8347, 0.9125)**	**0.6456 (0.6064, 0.6848)**
**Sensitivity**		
Internal CV	0.6604 (0.6597, 0.6611)	0.8447 (0.8437, 0.8456)
External validation	0.7742 (0.7616, 0.7868)	0.8881 (0.8626, 0.9135)
**Specificity**		
Internal CV	0.7418 (0.7412, 0.7425)	0.4715 (0.3415, 0.6014)
External validation	0.8601 (0.8496, 0.8706)	0.4032 (0.2562, 0.5502)
**Female**	**RF**	**Single threshold method**
**AUROC**		
**Internal CV**	**0.6665 (0.6596, 0.6733)**	**0.5478 (0.4933, 0.6022)**
**External validation**	**0.6938 (0.6298, 0.7579)**	**0.5138 (0.4550, 0.5726)**
**Sensitivity**		
Internal CV	0.5736 (0.5729, 0.5743)	0.8905 (0.8895, 0.8916)
External validation	0.6923 (0.6796, 0.7050)	0.9121 (0.8925, 0.9317)
**Specificity**		
Internal CV	0.6521 (0.6515, 0.6528)	0.2050 (0.0961, 0.3139)
External validation	0.6139 (0.6005, 0.6272)	0.1154 (0.0370, 0.1938)

Logistic Regression Model

Logistic regression is a simple but powerful method, especially for binary outcome. One key component is the logistic function, which could convert the multivariable input into the probability of the outcome between 0 and 1. Among all the machine learning algorithms, logistic regression has multiple advantages. First, no assumption is needed such as normal distribution of independent variables; Second, no assumption is needed about linear relationship between outcome and covariates. Most importantly, it is easy to understand and interpret the results.

Support Vector Machines (SVM) Model

SVM is another popular machine learning algorithm based on statistical learning theory. The SVM algorithm is to find a decision boundary which could maximize the distance between the two closest classes. The biggest advantage for SVM is that it could model non-linear decision boundary. It has multiple kernel functions and it is pretty robust against over fitting. However, one disadvantage to this algorithm is that SVM is very memory intensive and may not scale well to large datasets.

Random Forest Model

Random forests are considered as one of the most accurate machine learning methods, which are an ensemble classifier and proved to be the top winner in several data competitions. Random forests consist of many decision trees and combine the result from the individual trees. The attractive benefits using random forests lie in the following facts: 1) random forests could handle thousands of input variables without variable selection, which is heavy burden for logistic regression; 2) through large number of decision trees within random forest, it could produce an unbiased estimate of the generalization error; 3) it may allow large portion of missing data. We followed the general procedures for optimizing hyperparameters in Random Forest classification. The two key hyperparameters are number of trees (ntree) and number of variables randomly sampled as candidates at each split (mtry). We used the R package 'Caret' to support our optimization process. We created a list of different combination of mtry (from 1 to 8) and ntree=(from 50 to 1000 by 50), and compared the performance , and determined the best ntree and mtry. For the male model, ntree=500, mtry=3, for the female model, ntree=800, mtry=3.

All models were implemented in R 3.6.1.

© 2020 by the authors. Licensee MDPI, Basel, Switzerland. This article is an open access article distributed under the terms and conditions of the Creative Commons Attribution (CC BY) license (http://creativecommons.org/licenses/by/4.0/).
